# EMDR beyond PTSD: A Systematic Literature Review

**DOI:** 10.3389/fpsyg.2017.01668

**Published:** 2017-09-26

**Authors:** Alicia Valiente-Gómez, Ana Moreno-Alcázar, Devi Treen, Carlos Cedrón, Francesc Colom, Víctor Pérez, Benedikt L. Amann

**Affiliations:** ^1^Centre Emili Mira, Institute of Neuropsychiatry and Addictions, Parc de Salut Mar, Barcelona, Spain; ^2^Centre Fòrum Research Unit, Institute of Neuropsychiatry and Addictions, Parc de Salut Mar, Barcelona, Spain; ^3^Institut Hospital del Mar d'Investigacions Mèdiques, Barcelona, Spain; ^4^Department of Psychiatry, Autonomous University of Barcelona, Barcelona, Spain; ^5^Institute of Neuropsychiatry and Addictions Hospital del Mar, Parc de Salut Mar, Barcelona, Spain; ^6^Centro de Investigación Biomédica en Red de Salud Mental, Madrid, Spain

**Keywords:** eye movement desensitization and reprocessing, PTSD, psychosis, bipolar disorder, chronic pain, unipolar depression, RCT

## Abstract

**Background:** Eye Movement Desensitization and Reprocessing (EMDR) is a psychotherapeutic approach that has demonstrated efficacy in the treatment of Post-traumatic Stress Disorder (PTSD) through several randomized controlled trials (RCT). Solid evidence shows that traumatic events can contribute to the onset of severe mental disorders and can worsen their prognosis. The aim of this systematic review is to summarize the most important findings from RCT conducted in the treatment of comorbid traumatic events in psychosis, bipolar disorder, unipolar depression, anxiety disorders, substance use disorders, and chronic back pain.

**Methods:** Using PubMed, ScienceDirect, and Scopus, we conducted a systematic literature search of RCT studies published up to December 2016 that used EMDR therapy in the mentioned psychiatric conditions.

**Results:** RCT are still scarce in these comorbid conditions but the available evidence suggests that EMDR therapy improves trauma-associated symptoms and has a minor effect on the primary disorders by reaching partial symptomatic improvement.

**Conclusions:** EMDR therapy could be a useful psychotherapy to treat trauma-associated symptoms in patients with comorbid psychiatric disorders. Preliminary evidence also suggests that EMDR therapy might be useful to improve psychotic or affective symptoms and could be an add-on treatment in chronic pain conditions.

## Introduction

Eye Movement Desensitization and Reprocessing (EMDR) is a psychotherapeutic approach developed in the late 80s by Francine Shapiro (Shapiro, 1989) that aims to treat traumatic memories and their associated stress symptoms. This therapy consists of a standard protocol which includes eight phases and bilateral stimulation (usually horizontal saccadic eye movements) to desensitize the discomfort caused by traumatic memories and the aim of the therapy is to achieve their reprocessing and integration within the patient's standard biographical memories (Shapiro, 2005). The effectiveness of EMDR therapy in treating Post-traumatic Stress Disorder (PTSD) has undergone the scrutiny of several meta-analyses (Van Etten and Taylor, 1998; Bradley et al., 2005; Davidson and Parker, 2005; Seidler and Wagner, 2006; Benish et al., 2008; Jonas et al., 2013; Chen et al., 2014, 2015); this led to the final recognition by the World Health Organization (2013) as a psychotherapy of choice in the treatment of PTSD in children, teenagers, and adults^1^. Moreover, the application of EMDR therapy is not restricted to the treatment of people with PTSD and its use is currently expanding to the treatment of other conditions and comorbid disorders to PTSD (de Bont et al., 2013; Novo et al., 2014; Perez-Dandieu and Tapia, 2014). In this context, it is important to note that traumatic events belong to the etiological underpinnings of many psychiatric disorders (Kim and Lee, 2016; Millan et al., 2017). In addition, a comorbid diagnosis of PTSD can worsen the prognosis of other psychiatric disorders (Assion et al., 2009). Therefore, investigation in EMDR therapy has increased beyond PTSD and several studies have analyzed the effect of this therapy in other mental health conditions such as psychosis, bipolar disorder, unipolar depression, anxiety disorders, substance use disorders, and chronic back pain. The aim of this systematic and critical review is to summarize the most important results of the available randomized controlled trials (RCT) conducted in this field.

## Methods

**Figure 1:**
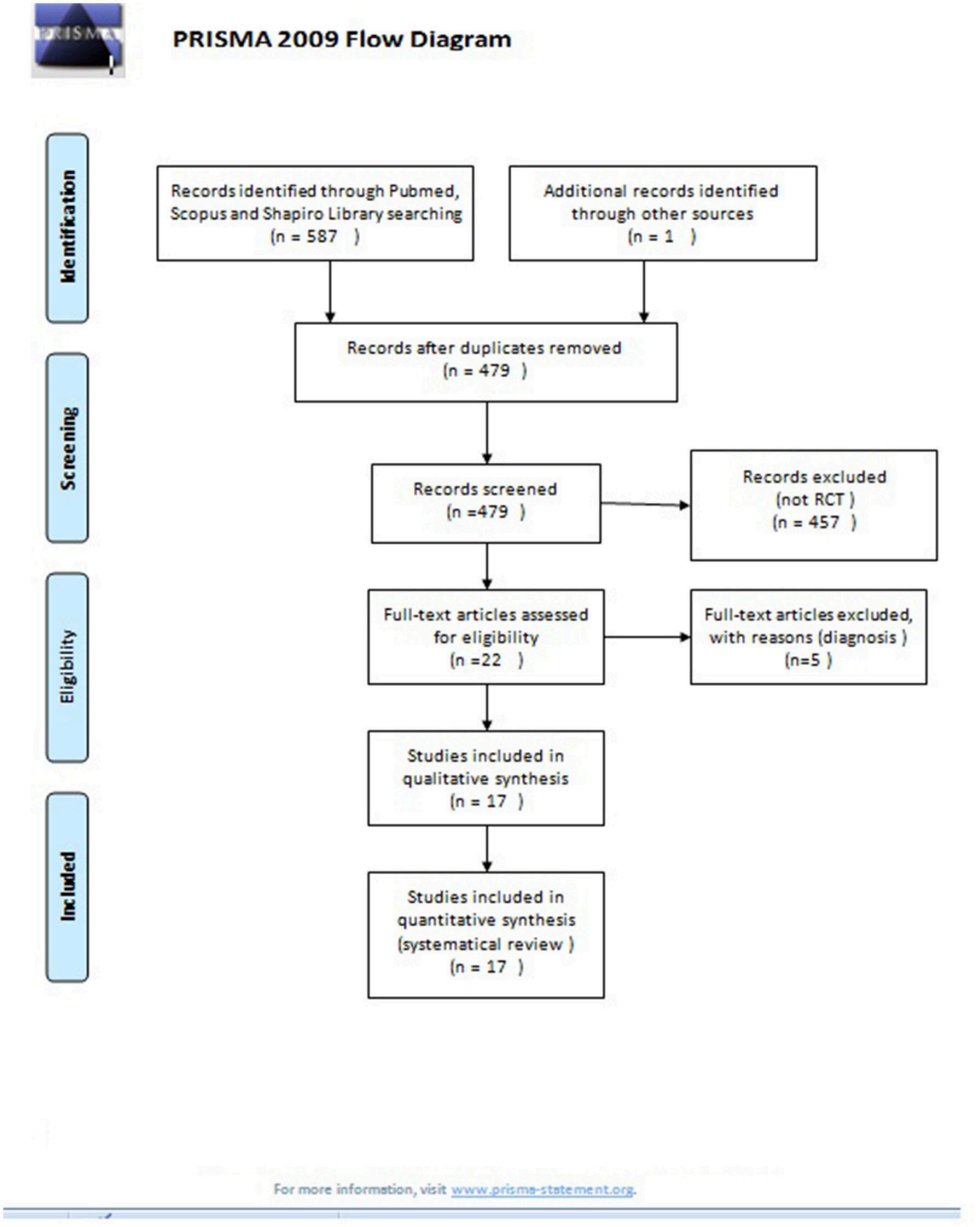
PRISMA 2009 flow diagram. From Moher et al. (2009).

Using PubMed, ScienceDirect, and Scopus, we conducted a systematic literature search of studies published up to December 2016, which examined the use of EMDR therapy in other psychiatric disorders beyond PTSD. The search terms were selected from the thesaurus of the National Library of Medicine (Medical Subject Heading Terms, MeSH) and the American Psychological Association (Psychological Index Terms) and included the terms “EMDR,” “schizophrenia,” “psychotic disorder,” “bipolar disorder,” “depression,” “anxiety disorder,” “alcohol dependence,” “addiction,” and “chronic pain.” The final search equation was defined using the Boolean connectors “AND” and “OR” following the formulation “EMDR” AND “schizophrenia”, “psychotic disorder,” “bipolar disorder,” “depression,” “anxiety disorder,” “alcohol or substance dependence” OR “addiction,” “chronic pain.” The automatic search was completed with a manual snowball search using reference lists of included papers and web-based searches in an EMDR-centered library (https://emdria.omeka.net/). The search included English-published articles from 01/01/1997 to 31/12/2016 and did not include any subheadings or tags (i.e., search fields “All fields”). Furthermore, we performed a manual search of the references list of previous meta-analysis and the retrieved articles. Case reports, serial cases, unpublished studies, and non-randomized studies, were excluded from this systematic review. Due to the significant heterogeneity of the studies, a formal quantitative synthesis (i.e., meta-analysis) was not possible. Instead, a systematic review was conducted using the PRISMA guidelines as referenced above. Prisma 2009 checklist (Supplementary Datasheet) and flow chart (Figure 1), as well as the Jadad scale (Supplementary Table) for reporting RCT have been completed and included in the Supplementary Material.

### Inclusion criteria and exclusion criteria

The final selection of the articles was carried out using the following criteria: (i) RCT published in peer-reviewed journals, (ii) in adult populations (over 18 years) that (iii) examined the use of EMDR therapy in different psychiatric disorders (as previously described). The criteria for exclusion were: (i) articles that did not contain original research (i.e., reviews and meta-analyses and (ii) quasi-experimental designs (single case and/or no control group). The studies were selected by Alicia Valiente-Gómez and discrepancies were resolved by Ana Moreno-Álcazar and Benedikt L. Amann.

## Results

### EMDR therapy in schizophrenia and other psychotic disorders

Since 2010, five datasets of RCT have been published in patients with a psychotic disorder and a comorbid PTSD or traumatic events (see Table 1) (Kim et al., 2010; de Bont et al., 2013, 2016; van den Berg et al., 2015; Van Minnen et al., 2016). These consist of two pilot studies (Kim et al., 2010; de Bont et al., 2013) and one large RCT (van den Berg et al., 2015) with two further subanalysis (de Bont et al., 2016; Van Minnen et al., 2016).

**Table 1:** RCT of EMDR in psychotic disorder.

**Title author, year**	**Sample (n)**	**EM/Full protocol**	**Control condition**	**Main findings**	**Conclusions**
Kim et al., 2010	45	EMDR	PR or TAU	EMDR = PR = TAU, but EMDR>PR>TAU in negative symptoms.	No differences within all groups, except of advantage of EMDR in negative symptoms.
de Bont et al., 2013	10	EMDR	PE or WL	PE = EMDR>WL in trauma symptoms.	PTSD patients with schizophrenia benefit from trauma-focused treatment approaches.
van den Berg et al., 2015	155	EMDR	PE or WL	EMDR = PE> WL in trauma symptoms.	Both trauma-focuses treatments are effective and safe to treat PTSD symptoms in patients with chronic psychotic disorders.
Van Minnen et al., 2016^*^	108	DS	NDS	DS = NDS in trauma symptoms.	Trauma-focused treatments for DS should not be excluded from these treatments.
de Bont et al., 2016^*^	155	EMDR	WL or PE	PE = EMDR>WL In paranoid thoughts. PE>EMDR>WL in depressive symptoms.	No differences within all groups, except of advantage of EMDR in paranoid thoughts and PE in depressive symptoms.

RCT, Randomized controlled trial; EMDR, Eye Movement desensitization and reprocessing; PR, progressive relaxation; TAU, treatment as usual; PE, Prolonged exposure; WL, wait-list control; PTSD, Post-Traumatic Stress Disorder; DS, Dissociative Subtype of PTSD; NDS, Non-Dissociative Subtype of PTSD.

* *These data sets corresponds to the clinical trial ISRCTN 79584912 of van den Berg et al. (2015)*.

A Korean group (Kim et al., 2010) carried out the first RCT including 45 acute schizophrenic inpatients. Patients were randomized to 3 weekly sessions of EMDR therapy (lasting 60 to 90 min) (*n* = 15), 3 weekly sessions of progressive muscle relaxation therapy (*n* = 15) (the first session lasted 90 min and the other two sessions lasted 60 min), and treatment as usual (TAU, *n* = 15). In the EMDR condition, the therapeutic treatment targets included stressful life events related with the current admission, traumatic incidents from childhood or adulthood, treatment-related adverse events (e.g., involuntary admission or seclusion), and the experience of distressing psychotic symptoms. All patients received TAU, which consisted of naturalistic psychopharmacological treatment, individual supportive psychotherapy, and group activities whilst being admitted. All groups showed an improvement of the symptomatic domains, which included psychotic, anxious, and depressive symptoms, measured by the Positive and Negative Syndrome Scale (PANSS), the Hamilton Depression Rating Scale (HAM-D), and the Hamilton Anxiety Rating Scale (HAM-A). The variance analysis (ANOVA), revealed a significant improvement over time in each of the treatment groups; however, there was no significant differences between treatment groups for the total PANSS (*F* = 0.73, *p* = 0.49), HAM-D (*F* = 0.41, *p* = 0.67), or HAM-A (*F* = 0.70, *p* = 0.51). Still, the effect size for negative symptoms was larger for the EMDR condition (0.60 for EMDR, 0.39 for PMR and 0.21 for TAU only, no significant differences).

A Dutch group published a small pilot RCT in patients with psychosis and PTSD in 2013 (de Bont et al., 2013). Patients were randomized to prolonged exposure (PE) (*n* = 5) or EMDR therapy (*n* = 5) to treat PTSD symptoms with a maximum of 12 weekly sessions of 90 min. The PTSD diagnosis was verified using the Clinical-Administered PTSD Scale (CAPS) and the Post-traumatic Stress Symptom Scale Self-Report (PSS-SR). All patients were assessed with the Psychotic Symptoms Rating Scale interview (PSYRATS) and the Green Paranoid Thoughts Scale (GPTS) for psychotic symptoms. The mixed-model showed that in the intention to treat analysis, both groups reached a significant decrease of PTSD symptoms during the treatment phase (*p* < 0.001, *r* = 0.64), this effect was maintained in the post-treatment phase (*p* < 0.001, *r* = 0.73) and in the 3 months follow up phase (*p* < 0.001). The same group conducted a large single-blind RCT including a sample of 155 outpatients with a psychotic disorder (schizophrenia or schizoaffective disorder) and a comorbid PTSD (van den Berg et al., 2015). Patients were randomized to three different groups (PE, EMDR, and Waiting-List Condition). Forty-seven patients were in the waiting-list condition (WL), for the other two conditions, PE (*N* = 53) and EMDR therapy (*N* = 55), patients received 8 weekly sessions of 90 min each. PTSD symptoms were evaluated with the CAPS, PSS-SR, and the Post-traumatic Cognitions Inventory (PTCI). The authors found that EMDR and PE therapy were both superior to the WL condition in reducing PTSD symptoms (PE effect size 0.78, *t* = −3.84, *p* = 0.001; EMDR effect size 0.65, *t* = −3.26, *p* = 0.001). No significant differences were detected between PE and EMDR therapy.

Two further subanalysis of the main study were published (de Bont et al., 2016; Van Minnen et al., 2016). The first subanalysis (de Bont et al., 2016) provided evidence, that the severity of paranoid thoughts assessed by GPTS, decreased in a significant way (PE *t* = −2.86, *p* = 0.005; EMDR *t* = −2.68, *p* = 0.008) and rates of remission for psychotic disorders increased for both treatment conditions in comparison to the WL arm (de Bont et al., 2016). In another secondary analysis with a subsample of 108 patients (Van Minnen et al., 2016), the authors evaluated the effectiveness of both trauma-focused treatment for patients with psychosis with and without the dissociative subtype of PTSD. This diagnosis was established regarding the items 29 (derealization) and/or 30 (depersonalization) (frequency ≥1 and intensity ≥2) on the CAPS. They though that, even though patients with a dissociative subtype of PTSD, showed significantly more severe PTSD symptoms at pre-treatment (*t* = −0.29, *p* = 0.005), the CAPS scores did no longer differ at post-treatment (*t* = −1.34, *p* = 1.85), when compared to patients without the dissociative subtype of PTSD.

In summary, one pilot study (Kim et al., 2010) found that EMDR therapy did not have a superior effect over progressive relaxation therapy or TAU in reducing trauma symptoms patients with PTSD and a psychotic disorder. In contrast, another preliminary study provided a comparable effect of EMDR therapy to PE (de Bont et al., 2013). This was confirmed by a large and well-designed study (van den Berg et al., 2015) that suggested that patients with a psychotic disorder and PTSD improved both with EMDR therapy and PE therapy (comparable to WL) in trauma-associated and paranoid symptoms, despite the impact and the high prevalence of comorbid PTSD in psychotic disorders, evidence of the use of EMDR therapy in psychosis and trauma is still scarce.

### EMDR therapy in affective disorders

#### EMDR therapy in bipolar disorder

So far, only 1 RCT has investigated the efficacy of EMDR therapy in bipolar disorder (Novo et al., 2014). Twenty bipolar patients with subsyndromal symptoms and a history of traumatic events were randomly assigned to 12 weeks of treatment with EMDR therapy or TAU. The participants were re-assessed at the end of this period and after a further 12 weeks of follow-up. Results showed significant reductions in affective scores in favor of the EMDR group after treatment. Affective symptoms were assessed through the HAM-D (*F* = 23.86, *p* = 0.001) and the Young Mania Rating Scale (YMRS) (*F* = 14.41, *p* = 0.004). However, changes from baseline to 24 weeks follow-up did not reach statistical significance. Regarding trauma symptoms, assessed by the CAPS and the Impact Event Scale (IES), results showed significant improvement in the EMDR group after treatment in both measures (CAPS *F* = 6.26, *p* = 0.03; IES *F* = 20.36, *p* = 0.001). At the follow-up assessment, only the IES scores remained statistically significant (*F* = 20.32, *p* = 0.003). Functional impairment was also assessed, but no group differences were found (Table 2).

**Table 2:** RCTs of EMDR in affective disorder, substance use disorders and chronic pain.

**Author, year**	**Diagnosis**	**Sample (n)**	**EM/Full protocol**	**Control condition**	**Main findings**	**Conclusions**
**AFFECTIVE DISORDERS**						
Novo et al., 2014	Bipolar disorder	20	EMDR	TAU	EMDR>TAU in trauma, depressive and hypomanic symptoms.	EMDR can help to treat subsyndromal mood beyond trauma symptoms in bipolar patients.
Hase et al., 2015	Unipolar depression	16	EMDR+TAU	TAU	EMDR+TAU>TAU	EMDR has positive effects in the treatment of depression.
Behnammoghadam et al., 2015	Depression after myocardial infarction	60	EMDR	WL	EMDR>WL	EMDR is an efficient treatment to depression in patients with myocardial infarction.
**SUBSTANCE USE DISORDERS**						
Hase et al., 2008	Alcohol Dependence	34	EMDR+TAU	TAU	EMDR+TAU>TAU	EMDR might be a useful approach for treating addiction memory and craving of alcohol.
Perez-Dandieu and Tapia, 2014	Alcohol and other substance use disorders	12	EMDR+TAU	TAU	EMDR+TAU>TAU	PTSD symptoms can be successfully treated with EMDR in substance abuse patients.
**CHRONIC BACK PAIN**						
Gerhardt, 2016		40	EMDR	TAU	EMDR>TAU	Pain-focused EMDR might be useful for non-specific chronic back pain patients.

*RCT, Randomized controlled trial; EMDR, Eye Movement desensitization and reprocessing; TAU, Treatment as usual; WL, waiting list*.

#### EMDR therapy in unipolar depression

Two controlled studies in EMDR therapy have been performed in unipolar depressive disorders (Behnammoghadam et al., 2015; Hase et al., 2015). A matched pairs study (Hase et al., 2015) was conducted with 32 inpatients currently suffering from mild-to-moderate depressive episodes related to recurrent depression according to the ICD-10 criteria. One group was treated with EMDR therapy (*N* = 16) in addition to TAU and matched by time of admission, gender and age with 16 controls who only received TAU. Usually, only one EMDR session was provided. In the case of an incomplete session, a second EMDR therapy session was added. EMDR therapy focused on disturbing memories related to the onset and course of the depressive disorder; however, most of the traumatic memories did not meet PTSD criteria. The TAU arm consisted of individual psychodynamic psychotherapy, group therapy sessions and five group sessions of psychoeducation. All patients were assessed by the Beck Depression Inventory (BDI), the Depression subscale of the Symptom Checklist 90 revised (SCL-90-R), and the SCL-90-R Global Severity Index (GSI). The authors found that TAU plus EMDR therapy was more effective than TAU by itself in reducing depressive symptoms [significant pre-post differences in SCL-90-R GSI score (*p* = 0.015) and in SCL-90-R Depression subscale score (*p* = 0.04)].

Regarding the second study, the efficacy of EMDR therapy on depression of patients with post-myocardial infarction was tested (Behnammoghadam et al., 2015). Sixty patients were randomized to EMDR therapy, receiving three sessions of 45–90 min per week during 4 months, or to a control group without any psychotherapeutic intervention. All participants were assessed by the BDI at the beginning and end of the study. The EMDR group showed significant differences in the depressive scores of the BDI before and after the EMDR therapy (27.26 ± 6.41 and 11.76 ± 3.71, *p* < 0.001). Mean scores of BDI also resulted significantly different between both groups at the end of the study (experimental group 11.76 ± 3.71 vs. control group 31.66 ± 6.09, *p* < 0.001). The authors concluded that EMDR therapy was an effective, useful, efficient and non-invasive method to treat depressive disorders in post-myocardial infarction patients (Table 2).

In summary, EMDR therapy has demonstrated preliminary positive evidence in one RCT as a promising therapy to treat depressive symptoms in unipolar depression (Hase et al., 2015). Furthermore, it might be a helpful tool to facilitate psychological and somatic improvement in patients with myocardial infarction who suffer subsequent depressive symptoms (Behnammoghadam et al., 2015).

### EMDR therapy in anxiety disorders

Six randomized studies have been carried out with EMDR therapy in anxiety disorders, beyond the diagnosis of PTSD (see Table 3) (Feske and Goldsteina, 1997; Goldstein et al., 2000; Nazari et al., 2011; Doering et al., 2013; Triscari et al., 2015; Staring et al., 2016).

**Table 3:** RCTs of EMDR in anxiety disorders.

**Author, year**	**Diagnosis**	**Sample (*n*)**	**EM/Full protocol**	**Control condition**	**Main findings**	**Conclusions**
Feske and Goldsteina, 1997	Panic disorder with agoraphobia	43	EMDR	WL or EFER	EMDR>WL in panic-related symptoms. EMDR = EFER	This study provides initial support for EMDR in the treatment for panic disorder.
Goldstein et al., 2000	Panic disorder with agoraphobia	45	EMDR	TAU and WL	EMDR = TAU>WL for anxiety, severity and agoraphobia. EMDR = WL < TAU for panic attack frequency and anxious cognitions.	EMDR partly effective but did not reduce panic attack frequency.
Doering et al., 2013	Dental phobia	31	EMDR	WL	EMDR>WL in dental anxiety and avoidance behavior.	EMDR effective in processing memories of past dental events in patients with dental phobia.
Triscari et al., 2015	Flying anxiety	65	EMDR+CBT	CBT-SD or CBT-VRET	EMDR+CBT = CBT-VRET = CBT-SD	Trauma focuses approaches are effective to treat patients with flying anxiety.
Staring et al., 2016	Anxiety disorders	47	EMDR	COMET	COMET>EMDR in self-esteem in anxiety disorders.	EMDR did not improve self-esteem in patients with anxiety disorders.
Nazari et al., 2011	OCD	90	EMDR	CTP	EMDR>CTP	EMDR can be more useful in short term than medication in improvement of OCD symptoms.

*RCT, Randomized controlled trial; EMDR, Eye Movement desensitization and reprocessing; WL, wait-list control; EFER, Eye fixation exposure and reprocessing; TAU, treatment as usual; CPT, Citalopram; BDORT, Bi-Digital-O-Ring-Test; CBT, Cognitive Behavioral therapy; CBT-SD, Cognitive Behavioral Therapy integrated with systematic desensitization; VRET, Cognitive Behavioral Therapy +virtual reality exposure therapy; COMET, Competitive Memory Training*.

The first study was carried out by Feske and Goldsteina (1997) in a sample of 43 patients with a diagnosis of panic disorder with agoraphobia. The diagnosis was established when symptoms were present for at least 1 year and at least one panic attack had occurred during the 2-week pre-test monitoring period. The subjects were randomized to EMDR therapy, eye fixation exposure and reprocessing therapy (EFER) (a version of EMDR omitting the ocular movements) or WL. The main aims of this study were to assess the efficacy of EMDR therapy in panic disorder and to analyze whether or not this correlates with the eye movements. Patients in both experimental groups, received five sessions over an average period of 3 weeks (one session of 120 min and four of 90 min). Authors found a significant improvement in post-treatment measures when comparing the EMDR group with the WL group (*p* < 0.05). ANCOVAS test revealed that the EMDR group was superior to the EFER group on 2 out of 5 primary measures of anxiety, specifically in the Agoraphobia-Anticipated Panic-Coping Composite (*F* = 7.65, *p* = 0.009) and General Anxiety-Fear of Panic Composite (*F* = 5.28, *p* = 0.028), on secondary measures of depression (BDI *F* = 4.96, *p* = 0.033), and on social adjustment, measured by the Social Adjustment Scale, Self-Report (*F* = 5.96, *p* = 0.020). However, at 3 months follow up, results did not remain significant.

Goldstein et al. aimed to replicate these results in 46 outpatients with a panic disorder and agoraphobia. Patients were randomized to EMDR therapy (6 sessions lasting 90 min conducted along 4 weeks), a credible attention-placebo control group or to a WL condition (Goldstein et al., 2000). The attention-placebo condition, consisted in a combination of 30–45 min of progressive muscle relaxation training and 45–60 min of association therapy. Compared to the WL condition, patients in the EMDR group showed a significant improvement on the measures of severity of anxiety, panic disorder and agoraphobia (*F* = 9.91, *p* ≤ 0.01), but the authors did not find significant changes in panic attacks frequency (*F* = 1.3, *p* ≥ 0.05) nor in anxious cognitions (*F* = 2.69, *p* ≥ 0.05). They found that EMDR therapy was superior to WL with a medium to large effect for all anxiety measures. ANOVAs test did not show any significant differences between EMDR therapy and the credible attention-placebo control condition (all measures: cognitive measures, panic and agoraphobic severity, diary and panic frequency were *p* > 0.13). Although EMDR therapy was superior to the WL condition, they concluded, based on their results, that EMDR therapy should not be the first-line treatment for panic disorder with agoraphobia.

One RCT so far has compared EMDR therapy with other psychotherapies to treat flight anxiety (Triscari et al., 2015). Of 65 patients, 22 patients were randomized to cognitive behavioral therapy integrated with systematic desensitization (CBT-SD), 22 patients to CBT with EMDR therapy (CBT-EMDR) and 21 patients to CBT combined with virtual reality exposure (CBT-VRET). All patients were assessed with the Flight Anxiety Situations Questionnaire and with the Flight Anxiety Modality Questionnaire. They received 10 weekly sessions of 2 h duration. No mean differences were found between the three groups after treatment or at follow-up, but all interventions showed efficiency in reducing fear of flying, demonstrating a high effect size (Cohen's *d* ranged from 1.32 to 2.23).

Another RCT has been performed in dental phobia (Doering et al., 2013). Sixteen patients were randomized to 3 weekly sessions of EMDR therapy, 90 min each, and 15 patients to a non-interventional WL. All patients were assessed with the Dental Anxiety Scale (DAS) and the Dental Fear Survey (DFS), secondary measures were assessed with the Brief Symptom Inventory and the Clinical Global Impression Score. Anxiety and depressive symptoms were assessed with the German Version of Hospital Anxiety and Depression Scale, symptoms of PTSD with the Impact of Event Scale-Revised and dissociative symptoms with the German Version of Dissociative Experiences Scale. The EMDR group demonstrated a significant decrease of dental anxiety scales with an effect size of 2.52 and 1.87 in DAS and DFS, respectively (*p* < 0.001). The effect sizes after 3 months (DAS 3.28 and DFS 2.28) and after 12 months (DAS 3.75 and DFS 1.79) persisted among the follow-up (*p* < 0.001). The most important result of this study was that a high number of patients overcame their avoidance behavior and visited the dentist regularly following treatment.

Furthermore, a recent trial compared EMDR therapy and competitive memory training (COMET) in the treatment of anxiety disorders with the purpose to improve self-esteem (Staring et al., 2016). The authors included 47 patients with a primary anxiety disorder and low self-esteem, which were assessed by the Rosenberg Self-esteem Scale, the Self-esteem Rating Scale-short Form and the STAI. Depressive symptoms were evaluated with BDI-II. Patients were randomized in a crossover design. Twenty-four patients received 6 EMDR therapy sessions and then 6 COMET sessions, the other 23 patients received firstly 6 COMET sessions and then 6 EMDR therapy sessions. COMET was more effective in improving self-esteem than EMDR therapy (effect sizes of 1.25 vs. 0.46, respectively). When EMDR therapy was applied before COMET, the effects of COMET on self-esteem and depression were significantly reduced. It could be hypothesized that EMDR therapy could diminish the effectiveness of the COMET intervention.

Finally, 1 RCT was performed in obsessive–compulsive disorder (OCD) (Nazari et al., 2011). They recruited a sample of 90 patients who were randomized to a treatment condition with Citalopram (a selective serotonin reuptake inhibitor) or EMDR therapy during 12 weeks. All subjects were assessed with the Yale-Brown Obsessive-Compulsive Scale before and after the treatment. They observed that both treatments were effective to treat obsessive symptoms, but the EMDR therapy group showed a faster improvement of obsessive and compulsive symptoms than the group treated with Citalopram (*p* = 0.001).

In summary, EMDR therapy has demonstrated in 4 RCT a positive effect on anxious and OCD symptoms (Feske and Goldsteina, 1997; Nazari et al., 2011; Doering et al., 2013; Triscari et al., 2015), whereas 1 RCT in panic disorder with agoraphobia was in part negative (Goldstein et al., 2000) and another study failed in improving self-esteem in patients with anxiety disorders (Staring et al., 2016).

### EMDR therapy in substance use disorders

Two studies so far have explored the efficacy of EMDR therapy in substance use disorders (Hase et al., 2008; Perez-Dandieu and Tapia, 2014). In a first study, 34 alcohol addicted patients were randomly assigned to TAU or TAU plus two sessions of EMDR therapy (Hase et al., 2008). The overall aim was to assess the craving intensity for alcohol via the Obsessive Compulsive Drinking Scale (OCDS) at pretreatment, post-treatment, and follow-up at 1 and 6 months. Likewise, other variables such as depression or anxiety symptoms were analyzed. Compared to pretreatment, post-treatment scores of craving and depression revealed a significant improvement in the experimental group (OCDS *t* = 10.7, *p* < 0.001; BDI *t* = 4.0, *p* = 0.001), while only a small reduction in both measures was noticed in the control group (OCDS *t* = 1.1, *p* = 0.29, BDI *t* = 0.9, *p* = 0.37). Between both groups, the difference in OCDS scores post-treatment was statistically significant (*p* < 0.001). These differences were maintained at 1-month follow-up (*p* < 0.05) but not at 6 months.

In a second study, 12 alcohol and/or drug addicted women with PTSD were randomized to TAU or TAU plus eight sessions of EMDR therapy (Perez-Dandieu and Tapia, 2014). Outcome criteria were PTSD symptoms, addiction symptoms, depression, anxiety, self-esteem [measured with Coopersmith's Self-esteem Inventory (SEI)] and alexithymia [assessed by Toronto Alexithymia Scale (TAS)]. Compared to pretreatment, PTSD scores showed a significant improvement in the experimental group compared to the control group (TAU+EMDR *t* = 4.22, *p* = 0.008; TAU *t* = −0.94, *p* = 0.38). Between both groups, the difference in the post-treatment PTSD scores, was also statistically significant (*p* < 0.01). Regarding addiction symptoms, no differences between both groups were detected. Finally, regarding the measures of depression, anxiety, self-esteem, and alexithymia, the experimental group showed a significant improvement in all of them except in the TAS (BDI *t* = 4.38, *p* = 0.007; STAI *t* = 2.65, *p* = 0.04; SEI *t* = −3.37, *p* = 0.01). On the contrary, the control group showed no significant differences in any measure. Between both groups, only the difference in post-treatment BDI scores were statistically significant (*t* = 14.13, *p* < 0.004).

Considering the results of both studies, EMDR therapy could be a useful therapy to use in substance use disorders with a history of traumatic life events in order to improve the prognosis of these patients (Perez-Dandieu and Tapia, 2014). Besides, EMDR therapy could help as an adjuvant psychotherapy to standard treatment of alcohol dependence directly decreasing craving (Hase et al., 2008; Table 2).

### EMDR therapy and chronic pain

One RCT has investigated so far the efficacy of EMDR therapy in the treatment of patients suffering from chronic pain (see Table 2; Gerhardt, 2016). Forty patients with chronic back pain and psychological trauma were randomized to 10 sessions of EMDR therapy in addition to TAU or TAU alone. The participants were re-assessed 2 weeks after study completion and also at 6 months follow-up after the end of the treatment. The primary outcome was its efficacy in pain reduction, measured by pain intensity, disability and treatment satisfaction. Estimated effect sizes between groups for pain intensity and disability were *d* = 0.79 (Ci_95%:_0.13, 1.42) and *d* = 0.39 (CI_95%:_−0.24, 1.01) at post-treatment and *d* = 0.50 (CI_95%:_0.14, 1.12) and *d* = 0.14 (Ci_95%:_−0.48, 0.76) at 6 months follow-up. Evaluation on treatment satisfaction from the patient's perspective showed that about 40% of the patients in the EMDR group in addition to TAU improved clinically and also rated their situation as clinically satisfactory, whilst in the control group, no patients showed clinical improvement. In view of these results, the authors concluded that EMDR therapy is a safe and effective therapeutic strategy to reduce pain intensity and disability in patients with chronic back pain.

## Discussion

This systematic review aimed to describe briefly the current evidence regarding EMDR therapy in patients with psychiatric conditions beyond PTSD but with a history of comorbid traumatic events. Even though RCT of EMDR therapy in severe mental disorders beyond PTSD are still scarce, an increased trend of publications at last decade has been observed. In general terms, we can conclude that there is currently insufficient evidence to recommend EMDR therapy as a treatment of choice in psychotic disorders and, so far, the same occurs with bipolar disorders (Kim et al., 2010; de Bont et al., 2013; Novo et al., 2014; van den Berg et al., 2015; Van Minnen et al., 2016). However, a large trial is being currently conducted in order to reach more accurate conclusions (Moreno-Alcazar et al, 2017).

The largest RCT of EMDR therapy in other psychiatric disorders has been performed in patients suffering from a psychotic disorder and a comorbid PTSD (van den Berg et al., 2015). Trauma-associated symptoms but also paranoid thoughts improved equally in both active comparators, EMDR and PE, when compared to WL. Both interventions were considered as safe. Both treatments were also effective in reducing PTSD symptoms with no significant differences between them in terms of effect or safety. The lack of superiority of EMDR therapy over the other treatment condition might be due to the fact that this study only applied 3 EMDR therapy sessions, which might be insufficient and infratherapeutic considering the symptomatic complexity of the sample, suffering from both schizophrenia and PTSD. In the subanalysis of the study, the authors pointed out that patients with a dissociative subtype of PTSD had a similar and favorable response to trauma focused treatments than those without the dissociative subtype, so this subgroup could benefit from this treatment and should not be excluded. These results are clinically relevant considering that patients with a psychotic disorder frequently suffer from comorbid adverse events/PTSD which affects in a negative way the course of the illness. Unfortunately, this is rarely taken into account when clinicians develop a personalized therapeutic plan, as therapists often believe treating traumatic events might deteriorate the patient's psychopathological state.

Similar to psychotic disorders, bipolar patients experience comorbid PTSD with a prevalence of 20% approximately (Hernandez et al., 2013; Passos et al., 2016; Cerimele et al., 2017). PTSD symptoms as well as life events cause more affective episodes (Simhandl et al., 2015). Therefore, trauma-orientated interventions need to be integrated in treatment strategies for bipolar patients. Positive evidence of trauma-orientated therapies, such as CBT and cognitive restructuring, exist in both psychotic and bipolar disorders with comorbid PTSD, these interventions have proven to be effective and safe (Mueser et al., 2008, 2015). Additionally, EMDR therapy has also been tested to treat traumatic symptoms in this population. Hereby in a pilot RCT including patients with a bipolar disorder (types I and II) with subsyndromal symptoms and a history of traumatic events, the authors found that patients showed an improvement in comparison to the TAU condition (Novo et al., 2014) and did not develop any mood episode related to the EMDR therapy. Given these results, EMDR therapy could be a promising and safe therapeutic strategy to reduce trauma symptoms and stabilize mood in traumatized bipolar patients, which is why a specific EMDR bipolar protocol has been suggested (Batalla et al., 2015). Currently, this EMDR protocol is being tested vs. supportive therapy in a large multicenter RCT including bipolar patients with a history of traumatic events (Moreno-Alcazar et al., 2017).

In depressive disorders, one study demonstrated the effectiveness of EMDR therapy compared to psychodynamic psychotherapy, group therapy, and psychoeducation therapy (Hase et al., 2008). EMDR therapy improved memories of stressful life events at onset of depressive episodes, emotional cognitive processing and long-term memory conceptual organization (Hase et al., 2008).

Within anxiety disorders, conflicting results were found in panic disorders with agoraphobia as it seems that EMDR therapy decreases severity of anxiety, panic disorder, and agoraphobia but not panic attacks frequency and anxious cognitions. Authors recommended EMDR therapy as an effective alternative to treat panic disorder with agoraphobia when other evidence-based treatments, such as exposure therapy or cognitive-behavior therapy, had failed. Nevertheless, panic disorder studies were not able to demonstrate an effect of EMDR therapy on anxious cognitions, as you would expect to find after applying the therapy. In OCD or phobias studies we did not find this fact. Further larger trials are needed to answer whether or not EMDR therapy is a valid therapeutic option as first line treatment in anxiety disorders and OCD.

Evidence of RCT so far suggests that EMDR therapy is a useful tool in the treatment of specific phobias, like flight anxiety or dental phobia, whether or not related to PTSD symptoms (Doering et al., 2013; Triscari et al., 2015).

In substance use disorders, EMDR therapy has been tested mainly in alcohol use disorders (Hase et al., 2008). EMDR therapy appears hereby to be useful as it decreases craving and drinking behavior (Hase et al., 2008; Perez-Dandieu and Tapia, 2014).

Finally, EMDR therapy was also effective in a first RCT for the treatment of chronic back pain (Gerhardt, 2016). This is not surprising as the impact of stress on both mental and physical health has been acknowledged for many years (Schneiderman et al., 2005). Pain as consequence of a traumatic event has been hereby identified as a risk factor for the development of PTSD (Norman et al., 2008) and often PTSD and chronic pain are concomitant (Beckham et al., 1997; Beck and Clapp, 2011; Moeller-Bertram et al., 2012). Again, further trials are needed to confirm the efficacy of EMDR therapy in this complex and often difficult to treat population.

The main limitation of this review is that RCT are scarce so far; however, as the use of EMDR therapy is increasing and gaining popularity, this systematic review is timely. Another limitation is that some of the included studies had very few therapeutic sessions. The high heterogeneity in number and duration of EMDR therapy sessions could have a negative effect on the results, so these must be taken cautiously (Hase et al., 2008, 2015; Kim et al., 2010; Behnammoghadam et al., 2015).

In general, EMDR therapy seems a safe intervention (Feske and Goldsteina, 1997; Hase et al., 2008, 2015; Doering et al., 2013; Novo et al., 2014; Perez-Dandieu and Tapia, 2014; Triscari et al., 2015; van den Berg et al., 2015; Gerhardt, 2016). This is of importance as it allows clinicians to consider EMDR therapy as an appropriate treatment in various psychiatric comorbid conditions without causing side effects.

## Author contributions

AV has performed the bibliographic search and has elaborated the first draft of the manuscript. AM has participated in the selection of included studies, resolved methodological doubts of possible studies, and helped in the first version of this manuscript. DT helped in the development of this review and revised the manuscript as native speaker. CC has collaborated in methodological aspects of this article. VP and FC have contributed in the improvement of the manuscript and BA had the idea of this work and revised the last version of this article.

### Conflict of interest statement

BA has been invited as speaker to several national and international EMDR congresses. VP has been a consultant or has received honoraria or grants from AstraZeneca, Bristol-Myers-Squibb, Janssen Cilag, Lundbeck, Otsuka, Servier, and Medtronic. The other authors declare that the research was conducted in the absence of any commercial or financial relationships that could be construed as a potential conflict of interest.

## Footnotes

^1^http://www.who.int/mediacentre/news/releases/2013/trauma_mental_health_20130806/es/2013.

## References

[B1] Assion H. J. Brune N. Schmidt N. Aubel T. Edel M. A. Basilowski M.. (2009). Trauma exposure and post-traumatic stress disorder in bipolar disorder. Soc. Psychiatry Psychiatr. Epidemiol. 44, 1041–1049. 10.1007/s00127-009-0029-1 19434346

[B2] Batalla R. Blanch V. Capellades D. Carvajal M. J. Fernández I. García F. (2015). EMDR therapy protocol for bipolar disorder, in Eye Movement Desensitization and Reprocessing (EMDR) Therapy Scripted Protocols and Summary Sheets: Treating Anxiety, Obsessive-Compulsive, and Mood-Related Conditions, ed Luber M. (New York, NY: Marilyn Luber), 223–287.

[B3] Beck J. G. Clapp J. D. (2011). A different kind of co-morbidity: understanding post-traumatic stress disorder and chronic pain. Psychol. Trauma 3, 101–108. 10.1037/a0021263 21765966 PMC3134278

[B4] Beckham J. C. Crawford A. L. Feldman M. E. Kirby A. C. Hertzberg M. A. Davidson J. R. (1997). Chronic post-traumatic stress disorder and chronic pain in Vietnam combat veterans. J. Psychosom. Res. 43, 379–389. 9330237 10.1016/s0022-3999(97)00129-3

[B5] Behnammoghadam M. Alamdari A. K. Behnammoghadam A. Darban F. (2015). Effect of Eye Movement Desensitization and Reprocessing (EMDR) on Depression in Patients With Myocardial Infarction (MI). Glob. J. Health Sci. 7, 258–262. 10.5539/gjhs.v7n6p258 26153191 PMC4803841

[B6] Benish S. G. Imel Z. E. Wampold B. E. (2008). Corrigendum to “The relative efficacy of bona fide psychotherapies for treating post-traumatic stress disorder: a meta-analysis of direct comparisons.” Clin. Psychol. Rev. 28, 766–775. 10.1016/j.cpr.2007.10.005 18055080

[B7] Bradley R. Greene J. Russ E. Dutra L. Westen D. (2005). A multidimensional meta- analysis of psychotherapy for PTSD. Am. J. Psychiatry 162, 214–227. 10.1176/appi.ajp.162.2.214 15677582

[B8] Cerimele J. M. Bauer A. M. Fortney J. C. Bauer M. S. (2017). Patients with co-occurring bipolar disorder and post-traumatic stress disorder: a rapid review of the literature. J. Clin. Psychiatry 78, e506–e514. 10.4088/JCP.16r10897 28570791

[B9] Chen L. Zhang G. Hu M. Liang X. (2015). Eye movement desensitization and reprocessing vs. cognitive-behavioral therapy for adult post-traumatic stress disorder: systematic review and meta-analysis. J. Nerv. Ment. Dis. 203, 443–451. 10.1097/NMD.0000000000000306 25974059

[B10] Chen Y. R. Hung K. W. Tsai J. C. Chu H. Chung M. H. Chen S. R. (2014). Efficacy of eye-movement desensitization and reprocessing for patients with post-traumatic-stress disorder: a meta-analysis of randomized controlled trials. PLoS ONE 9:e103676 10.1371/journal.pone.0103676 25101684 PMC4125321

[B11] Davidson P. R. Parker K. C. H. (2005). Eye movement desensitization and reprocessing (EMDR): a meta-analysis. J. Consult. Clin. Psychol. 69, 305–316. 10.1037/0022-006X.69.2.305 11393607

[B12] de Bont P. A. van den Berg D. P. G. van der Vleugel B. M. de Roos C. de Jongh A. van der Gaag M.. (2016). Prolonged exposure and EMDR for PTSD v. a PTSD waiting-list condition: effects on symptoms of psychosis, depression and social functioning in patients with chronic psychotic disorders. Psychol. Med. 46, 2411–2421. 10.1017/S0033291716001094 27297048

[B13] de Bont P. A. van den Berg D. P. van der Vleugel B. M. de Roos C. Mulder C. L. Becker E. S. (2013). A multi-site single blind clinical study to compare the effects of prolonged exposure, eye movement desensitization and reprocessing and waiting list on patients with a current diagnosis of psychosis and co morbid post traumatic stress disorder: study pro. Trials 23:151 10.1186/1745-6215-14-151 PMC3667059 23702050

[B14] de Bont P. A. Van Minnen A. De Jongh A. (2013). Treating ptsd in patients with psychosis: a within-group controlled feasibility study examining the efficacy and safety of evidence-based pe and EMDR protocols. Behav. Ther. 44, 717–730. 10.1016/j.beth.2013.07.002 24094795

[B15] Doering S. Ohlmeier M. C. de Jongh A. Hofmann A. Bisping V. (2013). Efficacy of a trauma-focused treatment approach for dental phobia: a randomized clinical trial. Eur. J. Oral Sci. 121, 584–593. 10.1111/eos.12090 24206075

[B16] Feske U. Goldsteina J. (1997). Eye movement desensitization and reprocessing treatment for panic disorder: a controlled outcome and partial dismantling study. J. Consult. Clin. Psychol. 65, 1026–1035. 10.1037/0022-006X.65.6.1026 9420364

[B17] Gerhardt A. (2016). Eye Movement Desensitization and reprocessing vs. treatment-as-usual for non-specific chronic back pain patients with psychological trauma: a randomized controlled pilot study. Front. Psychiatry 7:201. 10.3389/fpsyt.2016.00201 28066274 PMC5167699

[B18] Goldstein A. J. de Beurs E. Chambless D. L. Wilson K. A. (2000). EMDR for panic disorder with agoraphobia: comparison with waiting list and credible attention-placebo control conditions. J. Consult. Clin. Psychol. 68, 947–956. 10.1037/0022-006X.68.6.947 11142547

[B19] Hase M. Balmaceda U. M. Hase A. Lehnung M. Tumani V. Huchzermeier C.. (2015). Eye movement desensitization and reprocessing (EMDR) therapy in the treatment of depression: a matched pairs study in an inpatient setting. Brain Behav. 5:e00342. 10.1002/brb3.342 26085967 PMC4467776

[B20] Hase M. Schallmayer S. Sack M. (2008). EMDR Reprocessing of the addiction memory: pretreatment, posttreatment, and 1-month follow-up. J. EMDR Pract. Res. 2, 170–179. 10.1891/1933-3196.2.3.170

[B21] Hernandez J. M. Cordova M. J. Ruzek J. Reiser R. Gwizdowski I. S. Suppes T.. (2013). Presentation and prevalence of PTSD in a bipolar disorder population: a STEP-BD examination. J. Affect Disord. 150, 450–455. 10.1016/j.jad.2013.04.038 23706842

[B22] Jonas D. E. Cusack K. Forneris C. A. Wilkins T. M. Sonis J. Middleton J. C. (2013). Psychological and pharmacological treatments for adults with posttraumatic stress disorder (PTSD), in Psychol Pharmacol Treat Adults With Posttraumatic Stress Disord [Internet]. Available online at: http://www.ncbi.nlm.nih.gov/pubmed/23658937 23658937

[B23] Kim D. Choi J. Kim S. H. Oh D. H. Park S. Lee S. H. (2010). A pilot study of brief eye movement desensitization and reprocessing(EMDR) for treatment of acute phase schizophrenia. Korean J. Biol. Psychiatry 17, 94–102.

[B24] Kim J. S. Lee S. H. (2016). Influence of interactions between genes and childhood trauma on refractoriness in psychiatric disorders. Prog. Neuro Psychopharmacol. Biol. Psychiatry 70, 162–169. 10.1016/j.pnpbp.2016.01.013 26827636

[B25] Millan M. J. Ricca V. Oliver D. Kingdon J. Valmaggia L. McGuire P. (2017). Deconstructing vulnerability for psychosis: Meta-analysis of environmental risk factors for psychosis in subjects at ultra high-risk. Eur. Psychiatry 40, 65–75. 10.1016/j.eurpsy.2016.09.003 27992836

[B26] Moeller-Bertram T. Keltner J. Strigo I. A. (2012). Pain and post traumatic stress disorder - review of clinical and experimental evidence. Neuropharmacology 62, 586–597. 10.1016/j.neuropharm.2011.04.028 21586297

[B27] Moher D. Liberati A. Tetzlaff J. Altman D. G. The PRISMA Group (2009). Preferred reporting items for systematic reviews and meta-analyses: the PRISMA statement. PLoS Med. 6:e1000097. 10.1371/journal.pmed.1000097 19621072 PMC2707599

[B28] Moreno-Alcazar A. Radua J. Landin-Romero R. Blanco L. Madre M. Reinares M. (2017). Eye movement desensitization and reprocessing therapy vs. supportive therapy in affective relapse prevention in bipolar patients with a history of trauma: study protocol for a randomized controlled trial. Trials 18, 160 10.1186/s13063-017-1910-y 28376919 PMC5379519

[B29] Mueser K. T. Gottlieb J. D. Xie H. Lu W. Yanos P. T. Rosenberg S. D.. (2015). Evaluation of cognitive restructuring for post-traumatic stress disorder in people with severe mental illness. Br. J. Psychiatry 206, 501–508. 10.1192/bjp.bp.114.147926 25858178 PMC4450219

[B30] Mueser K. T. Rosenberg S. D. Xie H. Jankowski M. K. Bolton E. E. Lu W.. (2008). A randomized controlled trial of cognitive-behavioral treatment for posttraumatic stress disorder in severe mental illness. J. Consult. Clin. Psychol. 76, 259–271. 10.1037/0022-006X.76.2.259 18377122 PMC3916092

[B31] Nazari H. Momeni N. Jariani M. Tarrahi M. J. (2011). Comparison of eye movement desensitization and reprocessing with citalopram in treatment of obsessive-compulsive disorder. Int. J. Psychiatry Clin. Pract. 15, 270–274. 10.3109/13651501.2011.590210 22122001

[B32] Norman S. B. Stein M. B. Dimsdale J. E. Hoyt D. B. (2008). Pain in the aftermath of trauma is a risk factor for post-traumatic stress disorder. Psychol. Med. 38, 533–542. 10.1017/S0033291707001389 17825121

[B33] Novo P. Landin-Romero R. Radua J. Vicens V. Fernandez I. Garcia F.. (2014). Eye movement desensitization and reprocessing therapy in subsyndromal bipolar patients with a history of traumatic events: a randomized, controlled pilot-study. Psychiatry Res. 219, 122–128. 10.1016/j.psychres.2014.05.012 24880581

[B34] Passos I. C. Jansen K. de Cardoso T. A. Colpo G. D. Zeni C. P. Quevedo J.. (2016). Clinical outcomes associated with comorbid posttraumatic stress disorder among patients with bipolar disorder. J. Clin. Psychiatry 77, e555–e560. 10.4088/JCP.15m09935 27135375

[B35] Perez-Dandieu B. Tapia G. (2014). Treating trauma in addiction with EMDR: a pilot study. J. Psychoactive Drugs 46, 303–309. 10.1080/02791072.2014.921744 25188700

[B36] Schneiderman N. Ironson G. Siegel S. D. (2005). Stress and health: psychological, behavioral, and biological determinants. Annu. Rev. Clin. Psychol. 1, 607–628. 10.1146/annurev.clinpsy.1.102803.144141 17716101 PMC2568977

[B37] Seidler G. H. Wagner F. E. (2006). Comparing the efficacy of EMDR and trauma-focused cognitive-behavioral therapy in the treatment of PTSD: a meta-analytic study. Psychol. Med. 36, 1515. 10.1017/S0033291706007963 16740177

[B38] Shapiro F. (1989). Eye movement desensitization: a new treatment for post-traumatic stress disorder. J. Behav. Ther. Exp. Psychiatry 20, 211–217. 2576656 10.1016/0005-7916(89)90025-6

[B39] Shapiro F. (2005). Desensibilización y Reprocesamiento Por Movimiento Ocular, 2nd Edn. México: Pax.

[B40] Simhandl C. Radua J. Konig B. Amann B. L. (2015). The prevalence and effect of life events in 222 bipolar I and II patients: a prospective, naturalistic 4 year follow-up study. J Affect Disord. 170, 166–171. 10.1016/j.jad.2014.08.043 25240845

[B41] Staring A. B. P. van den Berg D. P. G. Cath D. C. Schoorl M. Engelhard I. M. Korrelboom C. W. (2016). Self-esteem treatment in anxiety: a randomized controlled crossover trial of Eye Movement Desensitization and Reprocessing (EMDR) vs. Competitive Memory Training (COMET) in patients with anxiety disorders. Behav. Res. Ther. 82, 11–20. 10.1016/j.brat.2016.04.002 27155451

[B42] Triscari M. T. Faraci P. Catalisano D. D'Angelo V. Urso V. (2015). Effectiveness of cognitive behavioral therapy integrated with systematic desensitization, cognitive behavioral therapy combined with eye movement desensitization and reprocessing therapy, and cognitive behavioral therapy combined with virtual reality expo. Neuropsychiatr. Dis. Treat. 11, 2591–2598. 10.2147/NDT.S93401 26504391 PMC4605250

[B43] van den Berg D. P. de Bont P. A. van der Vleugel B. M. de Roos C. de Jongh A. van d‘er Gaag M. (2015). Prolonged exposure vs eye movement desensitization and reprocessing vs waiting list for posttraumatic stress disorder in patients with a psychotic disorder a randomized clinical trial. JAMA Psychiatry 2, 259–267. 10.1001/jamapsychiatry.2014.2637 25607833

[B44] Van Etten M. L. Taylor S. (1998). Comparative efficacy of treatments for post-traumatic stress disorder: a meta-analysis. Clin. Psychol. Psychother. 5, 126–144. 10.1002/(SICI)1099-0879(199809)5:3<126::AID-CPP153>3.0.CO;2-H

[B45] Van Minnen A. Van der Vleugel B. M. Van der Berg D. P. G. de Bont P. de Roos C. van der Gaag M.. (2016). Effectiveness of trauma-focused treatment for patients with psychosis with and without the dissociative subtype of post-traumatic stress disorder. Br. J. Psychiatry 209, 347–348. 10.1192/bjp.bp.116.185579 27491533

