# Supplementary material for: EMDR beyond PTSD: A Systematic Literature Review
Source: Front Psychol. 2017 Sep 26;8:1668. doi: 10.3389/fpsyg.2017.01668 (PMC5623122; doi:10.3389/fpsyg.2017.01668)
Supplement: Supplementary file 1 [file Table1.docx]

| Study  (alphabetical order) | Randomization | Blinding | An account of all patients | Total score  (maximum points = 5) |
| --- | --- | --- | --- | --- |
| Behnammoghadam et al. (2015) | 1 | 0 | 1 | 2 |
| Dandieu et al. (2014) | 2 | 0 | 1 | 3 |
| De Bont et al. (2013) | 1 | 0 | 1 | 2 |
| De Bont et al. (2016) | 2 | 2 | 1 | 5 |
| Doering et al. (2013) | 2 | 2 | 1 | 5 |
| Feske and Goldstein. (1997) | 1 | 2 | 1 | 4 |
| Gerhardt et al. (2016) | 2 | 2 | 1 | 5 |
| Goldstein et al. (2000) | 1 | 0 | 1 | 2 |
| Hase et al. (2015) | 0 | 2 | 1 | 3 |
| Hase et al. (2008) | 0 | 0 | 1 | 1 |
| Kim et al. (2010) | 1 | 2 | 1 | 4 |
| Nazari et al. (2011) | 1 | 2 | 1 | 4 |
| Novo et al. (2014) | 1 | 2 | 1 | 4 |
| Staring et al. (2016) | 2 | 2 | 1 | 5 |
| Triscari et al. (2015) | 2 | 0 | 1 | 3 |
| Van den Berg et al. (2015) | 2 | 2 | 1 | 5 |
| Van Minnen et al. (2016) | 2 | 2 | 1 | 5 |

Table 6. Jadad scale for randomized controlled trials of EMDR in comorbid psychiatric disorders.
